# *On the Teneurin track*: a new synaptic organization molecule emerges

**DOI:** 10.3389/fncel.2015.00204

**Published:** 2015-05-27

**Authors:** Timothy J. Mosca

**Affiliations:** Department of Biology, Stanford UniversityStanford, CA, USA

**Keywords:** Drosophila, Teneurin, synapse development, synaptopathy, integrins

## Abstract

To achieve proper synaptic development and function, coordinated signals must pass between the pre- and postsynaptic membranes. Such transsynaptic signals can be comprised of receptors and secreted ligands, membrane associated receptors, and also pairs of synaptic cell adhesion molecules. A critical open question bridging neuroscience, developmental biology, and cell biology involves identifying those signals and elucidating how they function. Recent work in *Drosophila* and vertebrate systems has implicated a family of proteins, the Teneurins, as a new transsynaptic signal in both the peripheral and central nervous systems. The Teneurins have established roles in neuronal wiring, but studies now show their involvement in regulating synaptic connections between neurons and bridging the synaptic membrane and the cytoskeleton. This review will examine the Teneurins as synaptic cell adhesion molecules, explore how they regulate synaptic organization, and consider how some consequences of human Teneurin mutations may have synaptopathic origins.

## Introduction and History

The developing neuron has a myriad of tasks to complete along its path to become part of a functioning brain network. The final goal is to form a reliable synaptic connection with its defined partner. While synapse formation has been intensively studied (Waites et al., 2005; Craig and Kang, 2007; Dalva et al., 2007; McAllister, 2007; Colón-Ramos, 2009; Shen and Scheiffele, 2010; Siddiqui and Craig, 2011; Hruska and Dalva, 2012; Chia et al., 2013; Takahashi and Craig, 2013), a vast amount remains undetermined. Connections across the synaptic cleft are mediated by cell surface molecules, representing physical links between the pre- and postsynaptic membranes. Many of these molecules convey essential signals that coordinate the formation and function of synapses but our understanding of the identity of these molecules is incomplete. It is further unclear how these signals coordinate downstream events within the neuron like ordering the synaptic cytoskeleton, ensuring that release sites and neurotransmitter receptors properly align, and activating transcriptional networks in response to stimuli.

The Teneurins are a family of molecules that begins to answer some of these questions. Originally identified in *Drosophila* based on homology to Tenascin extracellular matrix proteins, they were first determined to be pair-rule genes: *ten-m* was named *odd oz* (*odz*) because of its expression in specific stripes of the fly embryo (Baumgartner and Chiquet-Ehrismann, 1993; Baumgartner et al., 1994; Levine et al., 1994). However, later analyses (Rubin et al., 1999; Tucker et al., 2012) revealed a resemblance to neuronal Tenascins, or Teneurins, also identified based on *ten-m* homology and diversely expressed in the brain, especially during development (Minet et al., 1999; Oohashi et al., 1999; Rubin et al., 1999, 2002; Tucker et al., 2000; Fascetti and Baumgartner, 2002; Zhou et al., 2003; Li et al., 2006; Kenzelmann et al., 2008).

**Figure 1:**
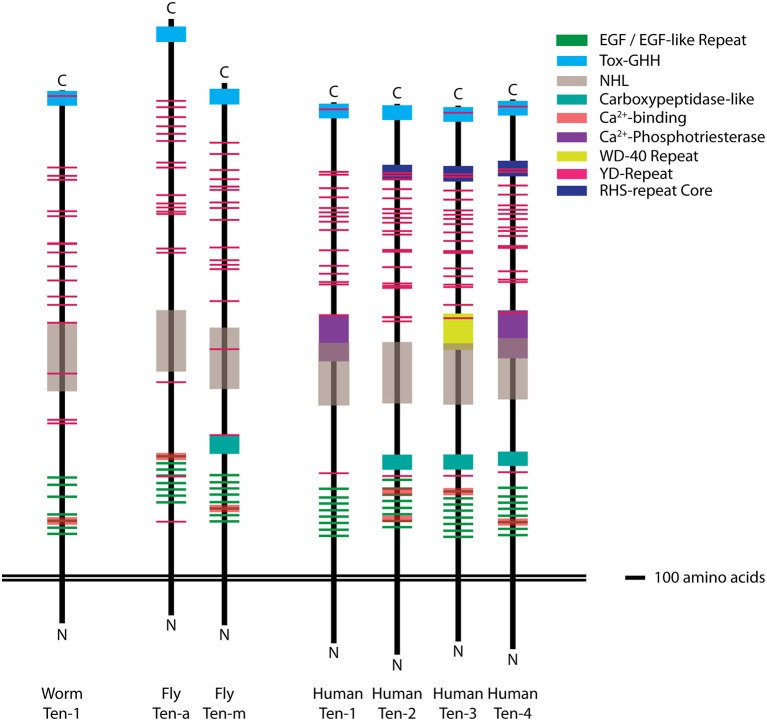
**Molecular structure of the Teneurins**. Diagram of the domain organization of the *C. elegans* Ten-1, the *Drosophila* Ten-m and Ten-a, and the human Ten-1, Ten-2, Ten-3, and Ten-4 proteins. The transmembrane domains are aligned as the reference point to facilitate comparison between the extracellular domains of each homolog. Across different species, the domain organization of the Teneurins is qualitatively similar and aligned at equivalent positions on the extracellular side. Domains were identified and mapped using NCBI sequences and domain prediction tools from SMART, Interpro, and NCBI. Each domain is color-coded (key) and scaled by size (scale = 100 amino acids). The NHL (gray) and Ca^2+^-binding (red) domains are shown at 65% transparency so as to indicate the dimensions of other, frequently overlapping, domains. Top = extracellular, Bottom = intracellular.

The Teneurins are large, type II cell surface proteins with a single transmembrane domain (Figure 1) and large extracellular C-termini with YD- and EGF-repeats for protein-carbohydrate and protein-protein interactions, respectively (Tucker and Chiquet-Ehrismann, 2006). Recent work suggests that the N-terminus participates in transcriptional regulation (Schöler et al., 2015). The Teneurins are conserved in many higher eukaryotes, with one homolog in *C. elegans*, two in *Drosophila*, and four each in most vertebrates (Figure 1; Tucker et al., 2012). Some possess Ca^2+^-dependent binding domains, as well as other known functional domains, the majority of which have undefined functions in Teneurin biology. Based on *in vitro* assays, vertebrate Teneurins form homo- and heterotypic dimers (Feng et al., 2002; Rubin et al., 2002): the strength of which can be mediated by the NHL domain (Beckmann et al., 2013). In the last decade, the Teneurins have emerged with multiple roles in the neuronal wiring between diverse pre- and postsynaptic partners. To ensure proper connectivity, neurons must select the proper area to project to (laminar specificity/region selection), identify the proper partner within that region (partner matching/cellular specificity), form robust connections with that partner (synapse formation and differentiation), and ensure that those connections persist (synaptic maintenance). Partner matching can be considered the last step of neuronal wiring, enabling the neuron to recognize its final target, before the growth cone undergoes morphological shifts to enable synapse formation (Kolodkin and Tessier-Lavigne, 2011). In *Drosophila*, the Teneurins mediate partner matching between select pre- and postsynaptic olfactory neurons as well as presynaptic motoneurons and postsynaptic muscles (Hong et al., 2012; Mosca et al., 2012) via a transsynaptic homophilic interaction. In vertebrate systems, the Teneurins are likely responsible for analogous processes ensuring proper visual connectivity (Leamey et al., 2007; Dharmaratne et al., 2012; Antinucci et al., 2013; Carr et al., 2013, 2014; Merlin et al., 2013; Young et al., 2013). This likely also occurs homophilically, though a heterophilic mechanism cannot currently be ruled out. As the roles for the Teneurins in partner matching and cellular specificity have been excellently examined elsewhere (Leamey and Sawatari, 2014 and others), this review will focus on a burgeoning role for synaptic Teneurins after neuronal wiring. These diverse activities include synapse induction, the precise alignment of active zones with postsynaptic receptors, pre- and postsynaptic differentiation, morphology regulation, recruitment of vesicles, signaling molecules and complexes, and the arrangement of a cytoskeletal meshwork to ensure spatial organization. This suite of exquisitely complex events can be broadly considered as synaptic organization. This review will consider the evidence, to date, for the Teneurins’ role in synaptic organization and how they may function to achieve that goal.

## Synaptic Teneurins in the *Drosophila* Central and Peripheral Nervous Systems

As neuronal cell surface molecules, the Teneurins are ideally poised to interact across the synaptic cleft. Historically, the neuromuscular junction (NMJ) has been the most frequently studied synapse in *Drosophila* due to its accessibility, simplicity, and available reagents for its molecular dissection (Collins and DiAntonio, 2007). Both *Drosophila* Teneurins, Ten-a and Ten-m, were implicated in NMJ synaptogenesis (Liebl et al., 2006; Kurusu et al., 2008) but not extensively examined until recently. Directed studies revealed that presynaptic Ten-a and postsynaptic Ten-m interact transsynaptically and heterophilically (Mosca et al., 2012). This drew a critical distinction between the Teneurins that regulate synaptic organization vs. partner matching. Teneurins are capable of homo- and heterophilic interactions (Feng et al., 2002; Silva et al., 2011; Beckmann et al., 2013; Boucard et al., 2014), where heterophilic interaction is defined as interacting with another partner or another Teneurin. In partner matching, the Teneurins are thought to function homophilically (Rubin et al., 2002; Leamey et al., 2007; Dharmaratne et al., 2012; Hong et al., 2012; Mosca et al., 2012; Antinucci et al., 2013; Carr et al., 2013, 2014; Merlin et al., 2013; Young et al., 2013) while synaptic organization occurs heterophilically (Silva et al., 2011; Mosca et al., 2012; Mosca and Luo, 2014).

**Figure 2:**
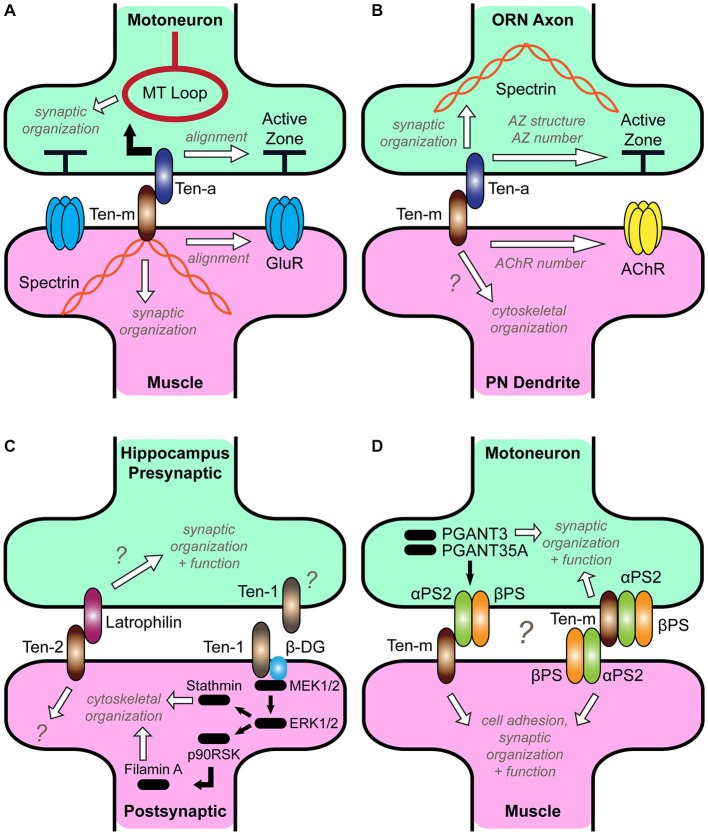
**Roles for Teneurins at diverse synapses. (A)** Teneurin function at the *Drosophila* neuromuscular junction (NMJ). Ten-a in the presynaptic motoneuron and Ten-m in the postsynaptic muscle interact transsynaptically to organize the cytoskeleton and ensure active zone apposition with glutamate receptors (Mosca et al., 2012). **(B)** Teneurin function in the *Drosophila* CNS. Ten-a in presynaptic olfactory receptor axons interacts transsynaptically with Ten-m in postsynaptic projection neuron dendrites to organize the spectrin cytoskeleton and ensure proper active zone and acetylcholine receptor number (Mosca and Luo, 2014). It is unknown whether postsynaptic Ten-m also regulates cytoskeletal organization (question mark). **(C)** Two models of Teneurin function in hippocampal neurons. On the left, Teneurin-2 is a postsynaptic receptor for Latrophilin (Silva et al., 2011). The downstream mechanisms that ensure synaptic organization and function on both sides remain unknown (question marks). On the right, Ten-1 interacts with β-Dystroglycan (β-DG) to activate a MEK/ERK pathway resulting in cytoskeletal rearrangement (Chand et al., 2012). The source of Ten-1, is unknown; though hypothesized to be postsynaptic, it could also be presynaptic (question mark). **(D)** A potential model for Teneurin signaling with integrins at the *Drosophila* NMJ. Ten-m and αPS2 interact (Graner et al., 1998), and PGANT3 and PGANT35A regulate Ten-m and integrin levels, leading to normal synaptic function (Dani et al., 2014). Ten-m has a minor presynaptic role (Mosca et al., 2012), and could interact with αPS2 either in *cis* or *trans*; as such, both models are presented (question mark). It is currently unclear how these interactions enable cell adhesion, synaptic organization, and function.

In *Drosophila*, Teneurins have two distinct expression levels. They are highly expressed at connections between *select* pairs of pre- and postsynaptic partners (Hong et al., 2012; Mosca et al., 2012). These levels are high during partner matching and persist after the connection has formed, suggesting a subsequent role in maintenance. This expression follows a homophilic pattern where pre- and postsynaptic partners express the same Teneurin. Secondly, at *all* neuromuscular and olfactory connections, a lower, basal level of expression exists, suggesting a more general role. Here, the interaction is heterophilic between presynaptic Ten-a and postsynaptic Ten-m. Perturbation of either component of this basal level at the NMJ causes a myriad of phenotypes including fewer synaptic boutons, failed active zone apposition, disorganization of synaptic proteins, failed pre- and postsynaptic differentiation, and reduced function (Mosca et al., 2012). These phenotypes are consistent with broad failures of synaptic organization and are present as soon as synapses begin forming (Mosca et al., 2012), suggesting an early involvement in synaptic organization, rather than maintaining formed connections. Disruption of heterophilic Teneurin interactions further caused marked disorganization of the presynaptic microtubule and the postsynaptic spectrin cytoskeletons. As the Teneurins interact with the cytoskeleton (Rubin et al., 2002; Nunes et al., 2005; Al Chawaf et al., 2007; Mörck et al., 2010; Zheng et al., 2011; Chand et al., 2012; Suzuki et al., 2014), this suggested that the Teneurins could (at least partially) exert their effects on diverse aspects of synaptic organization by ordering the cytoskeleton. Indeed, postsynaptic Ten-m interacts in a biochemical complex with muscle α-spectrin (Mosca et al., 2012), supporting this hypothesis. This demonstrated that a heterophilic Teneurin interaction enabled synaptic organization, likely by linking the neuronal membrane and the cytoskeleton (Figure 2A). Thus, cytoskeletal order is likely needed early to ensure synaptic organization. Thus, some synapses use Teneurins dually, as a homophilic partner matching signal and a heterophilic regulator of synaptic organization.

Both the olfactory system and the NMJ in *Drosophila* use Teneurins to ensure proper partner matching (Hong et al., 2012; Mosca et al., 2012). Could the same be said of their role in regulating synaptic organization? Using fluorescently labeled synaptic proteins (Fouquet et al., 2009; Leiss et al., 2009) and newly developed strategies for studying CNS synaptic organization (Kremer et al., 2010; Christiansen et al., 2011; Chen et al., 2014a; Mosca and Luo, 2014), the Teneurins were shown to mediate the proper density and structure of synapses in olfactory receptor neurons (Mosca and Luo, 2014). Teneurin perturbations in the CNS similarly reduced presynaptic active zones and postsynaptic acetylcholine receptor clusters. Strikingly, Teneurin CNS function is similar to that at the NMJ: Ten-a functions in presynaptic olfactory receptor neurons through its heterophilic partner Ten-m in postsynaptic projection neurons. Moreover, Ten-a functions upstream of, and in the same genetic pathway as, spectrin, supporting a model (Figure 2B) where Teneurins regulate cytoskeletal order in the CNS, which ensures the proper active zone number, structure, and spacing (Mosca and Luo, 2014).

These findings revealed a number of facets about Teneurin biology, synaptic regulation, and the logic underlying neuronal development. First, it identified the Teneurins as novel, critical components of the transsynaptic cadre of signals. Ten-a and Ten-m regulate cytoskeletal organization and cooperate with known transsynaptic signals like Neurexin/Neuroligin (Mosca et al., 2012) to coordinate synaptic organization. Second, the data revealed remarkable molecular conservation between partner matching and synaptic organization (Hong et al., 2012; Mosca et al., 2012; Mosca and Luo, 2014). The same molecules are used via different strategies (homo- vs. heterophilic binding) and expression to mediate their specific effects. Instead of evolving different mechanisms to achieve two goals, the nervous system adaptively reuses the same pathway using high homophilic Teneurin levels to enable partner matching while basal heterophilic Teneurin levels enable synaptic organization. Third, this highlights conservation between both peripheral and central nervous system processes. These two synapses (neuromuscular vs. olfactory) have distinct requirements (Wu et al., 2010; Benson and Huntley, 2012); as such, there is no inherent expectation for similarity in their governing principles. However, the shared cytoskeletal role for the Teneurins highlights conservation, allowing one molecule to achieve multiple developmental goals.

## Synaptic Teneurins in Vertebrate Systems

Recent evidence further suggests synaptic roles for Teneurins in diverse vertebrate systems. An unbiased proteomics screen (Silva et al., 2011) identified Teneurin-2 as the ligand for Latrophilin, a neuronal G-protein-coupled receptor (Südhof, 2001). The two proteins form a high-affinity, transsynaptic, heterophilic complex, localize to, and can induce synapses in hippocampal or artificial synapse cultures (Silva et al., 2011). Teneurin-2 interacts with Latrophilin (Figure 2C) via a region containing its highly conserved Tox-GHH domain (Figure 1), suggesting that this domain may be critical for function or adhesion (Zhang et al., 2012). Teneurin-4 can also interact with Latrophilin-1 (Boucard et al., 2014), further supporting heterophilic synaptic interactions (Mosca et al., 2012; Mosca and Luo, 2014). The functional significance of these interactions, however, is unclear. Application of the Teneurin-2 C-terminus in hippocampal culture induces presynaptic Ca^2+^ signaling (Silva et al., 2011). This is reminiscent of the Teneurin C-terminal associated peptide (TCAP), a 41-residue peptide that can be liberated and is thought to regulate stress response, dendritic remodeling in the hippocampus, BDNF levels, and corticotropin-releasing factor signaling (Rotzinger et al., 2010; Ng et al., 2012; Tan et al., 2012; Chen et al., 2013). Indeed, Teneurin-2 can be cleaved in culture (Silva et al., 2011), supporting this model but could also function by another, unknown mechanism. Application of the recombinant extracellular domain of Latrophilin-1 to cultured neurons can alternatively decrease synapse density (Boucard et al., 2014), but it is unclear whether this occurs via the Teneurins or by sequestration of another Latrophilin ligand.

Latrophilins regulate excitatory synapse development and strength *in vivo* (O’Sullivan et al., 2012, 2014). Latrophilin-3 interacts via its Olfactomedin and Lectin domains with Teneurin-1 and disruption of the Latrophilin-3 Olfactomedin domain impairs synaptic density (O’Sullivan et al., 2014). Moreover, Olfactomedin1 itself (whose binding targets include Teneurin-4) interacts with known synaptic proteins; its deletion causes brain dystrophy and behavioral phenotypes (Nakaya et al., 2013). This raises the possibility that Teneurin interaction with Olfactomedin-domain containing proteins is involved in ensuring synaptic protein localization and function. However, Latrophilin-3 also interacts with fibronectin leucine rich transmembrane (FLRT) proteins via this domain, which also mediate synaptic connectivity and function (O’Sullivan et al., 2012). Teneurins may also regulate synaptic function in the visual system, beyond their role for wiring (Leamey and Sawatari, 2014). Zebrafish *teneurin-3* knockdown impairs the morphology and connectivity of retinal ganglion cells (Antinucci et al., 2013) and these animals fail to develop proper orientation selectivity. It is unclear whether a role for Teneurin-3 in ensuring correct connectivity (i.e., via partner matching and wiring) can account for such deficits. Teneurin-3 mediated wiring of the visual system in zebrafish (Antinucci et al., 2013) could secondarily result in functional deficits. However, there could also be a role for Teneurin-3 in forming and maintaining the actual connections after partner matching. While further work is needed to determine the Latrophilin-3 ligand and the mechanism for failed direction selectivity following *teneurin-3* knockdown, the role of Teneurins at vertebrate synapses remains an exciting question of great importance for future work.

## Teneurins and the Symphony of Cell Surface Molecules at the Synapse

The Teneurins are one of a critical suite of cell surface molecules for synapse formation, organization, and function. These include cell adhesion molecules, ligand-receptor complexes, and secreted factors (Johnson-Venkatesh and Umemori, 2010; Yang et al., 2014). Though many signals are yet unknown, the intricacy and complexity of the synaptic landscape is clear (Cheng et al., 2006; Collins et al., 2006; Dahlhaus et al., 2011; Weingarten et al., 2014). Their natures are also remarkably diverse: they provide structural adhesion to ensure membrane apposition and facilitate neurotransmission. They convey developmental signals, ensuring that synaptic components are recruited at the proper time to the proper site. They enable synapses to respond to stimuli, enabling activity-dependent structural and functional changes and allowing for synaptic plasticity. Such cell surface molecules, are not all simply “glue” that holds synaptic membranes together. Even molecules like cadherins, neural cell adhesion molecules (NCAMs), L1CAMs, Nectins, Contactins, and synaptic adhesion-like molecules (SALMs), long classified as structural adhesion molecules can influence synaptic differentiation and recruit essential factors to synaptic contacts (Chen et al., 2014b; Mori et al., 2014; Yang et al., 2014; Friedman et al., 2015). In many cases, individual molecules fill a combination of these roles, defying classification into simple categories.

The Teneurins are synaptic cell surface molecules with such diverse functions. They are synaptic organizers (Scheiffele et al., 2000) as they can induce synapses on co-cultured cells (Silva et al., 2011). This role is shared with a number of other molecules. Neurexin and Neuroligin are the most studied synaptic organizer with links to neurodevelopmental disorders (Südhof, 2008). Neurexins, however, can mediate synaptic organization via other proteins including the leucine rich repeat transmembrane molecules (LRRTMs) and GluRδ2 (de Wit et al., 2009; Matsuda et al., 2010; Siddiqui et al., 2010; Uemura et al., 2010). These are not exclusive, however, as both the Neuroligins and LRRTMs are capable of interacting with additional proteins to organize synaptic contacts (Linhoff et al., 2009; Siddiqui and Craig, 2011; Siddiqui et al., 2013; de Wit et al., 2013). Additional synaptic organizers include the Ephrins and Eph receptors (Dalva et al., 2007; Hruska and Dalva, 2012), SynCAMs (Biederer et al., 2002), Protein Tyrosine Phosphatases (PTPs; Takahashi et al., 2011, 2012; Takahashi and Craig, 2013), and secreted factors like Wnts (Hall et al., 2000; Packard et al., 2002; Sahores et al., 2010; Dickins and Salinas, 2013), Semaphorins (Koropouli and Kolodkin, 2014), Thrombospondins (Christopherson et al., 2005; Eroglu et al., 2009), and fibroblast growth factors (FGFs; Umemori and Sanes, 2008; Terauchi et al., 2010). These all play roles in ensuring synapse formation, differentiation of pre- and postsynaptic machinery, and synaptic function (Siddiqui and Craig, 2011; Yang et al., 2014) though the specific mechanisms for many remain an active research question.

Beyond synaptic induction, the Teneurins also ensure an ordered cytoskeleton, a role which may be more unique to this family. While Nectins can organize actin (Mori et al., 2014) and Wnt signals regulate the synaptic cytoskeleton (Packard et al., 2002; Miech et al., 2008; Varela-Nallar et al., 2012; Lüchtenborg et al., 2014), mutation of genes like Neurexin and Neuroligin have little to no effect on the synaptic cytoskeleton (Li et al., 2007; Banovic et al., 2010; Mosca et al., 2012). An accurate, broad comparison, however, has eluded the field. Often, the cytoskeleton is not assayed following perturbation of synaptic cell surface molecules, preventing direct comparison. Work in *Drosophila* has compared Teneurin and Neuroligin1 perturbations (Mosca et al., 2012), showing that Neuroligin mutations only minor cytoskeletal phenotypes but severe active zone apposition defects (Banovic et al., 2010). Teneurin mutations, on the other hand, cause severe cytoskeletal disruption with only minor apposition phenotypes (Mosca et al., 2012). To ensure redundancy in such an important process as synapse formation and organization, this system likely uses a general principle involving major and minor roles. A gene predominantly controls one aspect while having secondary, minor roles in other facets of synapse organization. For Teneurins, they are the primary mediator of cytoskeletal order but have minor roles in synapse induction, differentiation, and apposition. For Neuroligin, these roles are reversed. An important direction for future studies will be to examine how varied molecules interact with each other. What are their major roles? What are their minor roles? How do they cooperate to ensure a properly formed and functioning synapse? This will provide vast insight on how multiple signals are coordinated at synaptic contacts to ensure smooth neuronal development.

## How do Teneurins Regulate Synaptic Organization?

A critical question is how the Teneurins regulate such diverse processes as neuronal wiring, synapse organization, morphogenesis, and patterning. In *Drosophila*, only two Teneurins exist, but regulate all of these events (Baumgartner et al., 1994; Levine et al., 1994; Kinel-Tahan et al., 2007; Zheng et al., 2011; Hong et al., 2012; Mosca et al., 2012; Cheng et al., 2013; Mosca and Luo, 2014). The Teneurins likely recognize other cells based on Teneurin expression and differentiate between simultaneously occurring homo- and heterophilic pairs. In the fly nervous system (Hong et al., 2012; Mosca et al., 2012; Mosca and Luo, 2014), homo- and heterophilic Teneurin interactions can occur between the same two cells: how does presynaptic Ten-a differentiate partner matching with postsynaptic Ten-a from synapse organization with postsynaptic Ten-m (Hong et al., 2012; Mosca and Luo, 2014)? In vertebrate systems, four homologs exist, raising the number of potential pairs to 10. How does a connection know which pair to listen to in fulfilling its goal? The answer likely lies in differing downstream interacting proteins, heterophilic ligands, developmental expression, and physical Teneurin properties. To date, the mechanisms for how homophilic partner matching occurs are unknown: identifying the underlying signals and molecules remains an active question. The second portion of this review will examine the evidence to date on potential interactors, and offer a perspective on how these mechanisms may mediate synaptic organization. It will focus on the *Drosophila* NMJ, where Teneurins have been most mechanistically studied, but further consider translation. While the more complex vertebrate nervous systems may have evolved additional mechanisms for controlling synaptic organization absent in the fly, it is important to note the CNS and PNS conservation of Teneurins in the *Drosophila* (Mosca et al., 2012; Mosca and Luo, 2014). Using similar mechanisms to build synapses with different structural, physiological, and evolutionary requirements suggests that such strategies may be broadly applicable across different systems.

### Potential Effectors of Teneurins in Synaptic Organization

At the NMJ, the Teneurins function to organize the cytoskeleton and ensure properly apposed active zones. Muscle Ten-m interacts in a complex with α-spectrin (Mosca et al., 2012), suggesting a direct link to the synaptic membrane and a foundation upon which an ordered cytoskeleton can be built. This is not limited to spectrin, as Teneurins also regulate actin-regulating proteins and adaptors like WASp and Adducin (Mosca et al., 2012). Further, loss of a postsynaptic muscle actin and Teneurin perturbation result in similar phenotypes (disrupted spectrin network, reduced subsynaptic reticulum, unapposed active zones, and reduced synaptic transmission) and both display genetic interactions with Neurexin and Neuroligin regarding active zone alignment (Mosca et al., 2012; Blunk et al., 2014). If Teneurins similarly regulated actin, this could account for the observed phenotypes, though their direct interaction has yet to be shown. Indeed, the intracellular N-termini of the Teneurins contain known polyproline sequences (Tucker and Chiquet-Ehrismann, 2006) that can interact with SH3 domains and the cytoskeleton (Mayer, 2001; Benz et al., 2008), further poising the Teneurins as general links between the membrane and the cytoskeleton to regulate synaptic organization.

While spectrin is also presynaptic, the microtubule cytoskeleton instead is the predominant player in ensuring proper NMJ morphology and function (Hummel et al., 2000; Roos et al., 2000; Zhang et al., 2001). Teneurin perturbation results in catastrophic disruption of microtubule organization (Mosca et al., 2012), much like direct perturbation of microtubule binding proteins (Hummel et al., 2000; Roos et al., 2000; Pennetta et al., 2002). Interestingly, with Teneurin perturbation, the presynaptic spectrin cytoskeleton remains organized enough such that phenotypes associated with its loss (fewer membrane proteins, severely reduced function, and synaptic retractions) are not evident (Featherstone et al., 2001; Pielage et al., 2005; Massaro et al., 2009). Teneurins could directly (or via an intermediary) link microtubules to the membrane. The Ankyrins, large adaptor proteins, are critical at the NMJ in ensuring cytoskeletal organization and proper synaptic structure (Koch et al., 2008; Pielage et al., 2008; Lüchtenborg et al., 2014). Understanding the interplay of Teneurins, Ankyrin, and others will reveal how general cytoskeletal organization is regulated by the neuronal membrane.

Beyond the cytoskeleton, the Teneurins have an independent role in regulating active zone apposition and structure. Teneurin perturbation causes failures (Mosca et al., 2012) of active zone apposition, but these phenotypes are less severe than those resulting from loss of Neurexin and Neuroligin (Li et al., 2007; Banovic et al., 2010; Sun et al., 2011; Chen et al., 2012; Mosca et al., 2012). These apposition, and also ultrastructural, defects cannot accounted for by cytoskeletal impairment (Hummel et al., 2000; Roos et al., 2000; Viquez et al., 2006, 2009; Massaro et al., 2009). Further, *neuroligin* and *ten-a* mutants synergize, suggesting an alternative mechanism. Indeed, ultrastructural defects like detached and misshapen active zones following Teneurin perturbation (Mosca et al., 2012) resemble those associated with perturbation of an adapter protein, DSyd-1 (Owald et al., 2010), which functions through Neurexin and Neuroligin to foster synaptic alignment (Owald et al., 2012). Thus, Teneurins may signal through a similar mechanism, either through direct interaction with DSyd-1 or with an additional adaptor. For these reasons, dissecting the precise roles of the Teneurins is a challenging, but rewarding goal. In further understanding the domains (Figure 1) necessary for each function, we can compare different synapses across different organisms to understand general Teneurin mechanisms. At the fly NMJ, Ten-m and Neuroligin colocalize (Mosca et al., 2012); thus, both can use different mechanisms to ensure synapse formation at the same, precise location on the neuronal membrane.

### Regulating Synapses through Interaction with Non-Teneurin Ligands

Teneurins can interact heterophilically with other Teneurins (Oohashi et al., 1999; Feng et al., 2002) or non-Teneurin ligands. This ability first identified mammalian Teneurins as a potential synaptic organizer via its interactions with Latrophilin (Silva et al., 2011; Boucard et al., 2014). However, these studies did not examine the consequences of Teneurin loss at vertebrate synapses; thus, this is one of the most important next steps regarding vertebrate Teneurin function. Some mechanistic clues, however, can be taken from the consequences of Teneurin interaction with heterophilic Integrin and Dystroglycan ligands. Integrins are transmembrane receptors that transsynaptically bridge pre- and postsynaptic neurons (Clegg et al., 2003; Singhal and Martin, 2011) or mediate interactions with the extracellular matrix (Broadie et al., 2011). Integrins often act with the Dystroglycan-Dystrophin complex to stabilize synaptic components (Pilgram et al., 2010) as Laminin receptors (Henry and Campbell, 1999). Dystroglycans bridge the extracellular matrix with the cytoskeleton, linking actin to Laminins and Dystrophin (Ibraghimov-Beskrovnaya et al., 1992; Ervasti and Campbell, 1993), and also organize postsynaptic regions (Bogdanik et al., 2008; Waite et al., 2012). Some of these interactions have implications for synaptic organization. In *C. elegans*, the Teneurin *ten-1* interacts with the Integrin and Dystroglycan homologs *ina-1* and *dgn-1* and the prolyl 4-hydroxylase *phy-1* to regulate collagen IV and maintain basement membranes during embryonic development (Trzebiatowska et al., 2008; Topf and Chiquet-Ehrismann, 2011). In *ten-1 phy-1* double mutants, embryos display gross defects in epidermal development, body wall musculature, and enhanced lethality. These phenotypes also synergize with mutations in collagen IV, leading to a model whereby epidermal TEN-1 binds collagen IV in the basement membrane. In the absence of *phy-1*, collagen IV is improperly processed and fails to be secreted into the basement membrane to bind TEN-1, weakening the muscle structure. Similar defects and interactions also occur with Integrin and Dystroglycan mutations (Trzebiatowska et al., 2008). As all Teneurin homologs possess an NHL domain (Figure 1), which can interact with integrins (Löer et al., 2008), this suggests that such a mechanism could be conserved across species. In mice, there is further interplay between Teneurin and Dystroglycan, as the C-terminal region of Teneurin-1 colocalizes with Dystroglycan (Chand et al., 2012, 2014). In hippocampal cultures, application of this C-terminus regulates cytoskeletal organization (presumably through Dystroglycan) by increasing tubulin levels, actin polymerization, and filopodia length and rate of formation. This occurs by activating MAPK to phosphorylate the cytoskeletal proteins stathmin and Filamin A, leading to cytoskeletal reorganization (Figure 2C). Thus, Teneurin-1 is proposed to induce a signal beginning from Dystroglycan at the membrane and resulting in neurite elongation (Chand et al., 2012). In the testes, this interaction is conserved, as Teneurin-1 colocalizes with actin, regulating testosterone and testicular size (Chand et al., 2014). This conserved Teneurin-Dystroglycan interplay is thus poised to generally regulate the cytoskeleton.

In *Drosophila*, synaptic Integrins and Dystroglycan regulate NMJ development (Hoang and Chiba, 1998; Beumer et al., 1999, 2002; Rohrbough et al., 2000; Bogdanik et al., 2008; Wairkar et al., 2008; Tsai et al., 2012a). Recent work has further linked this role to the Teneurins in identifying *pgant3* and *pgant35A*, two protein alpha-N-acetylgalactosaminyltransferases that regulate integrins. Mutations in these genes control the levels of the synaptic integrin receptor αPS2 and Ten-m (Dani et al., 2014); in their absence, synaptic Ten-m levels were reduced and rescued by neuronal restoration of either *pgant*. Further supporting this interplay is evidence that Ten-m and αPS2 directly interact (Graner et al., 1998). If αPS2 glycosylation maintains proper Ten-m levels, in its absence, Ten-m is downregulated. This is analogous to observed regulation of postsynaptic Ten-m by presynaptic Ten-a (Mosca et al., 2012). Ten-m and αPS2 interactions altered cell motility, suggesting they can regulate morphogenesis and outgrowth. At the NMJ, removal of presynaptic Ten-m modestly reduces the number of synaptic boutons via an unknown postsynaptic ligand (Mosca et al., 2012). Because of its ability to interact in *trans* (Graner et al., 1998) and its postsynaptic expression (Beumer et al., 1999), αPS2 may be this ligand, suggesting a potential model (Figure 2D). Further experiments will tease apart the precise roles for all partners. Such an interaction, though, would further intertwine the Teneurins with other synaptic signals. There is clear redundancy amidst signals (Craig and Kang, 2007; Dalva et al., 2007; Siddiqui and Craig, 2011; Chia et al., 2013; Takahashi and Craig, 2013; Yang et al., 2014); a critical goal will be to determine how they act in concert and identify the extent and nature of their specificity and redundancy.

### Differential Function via Expression and Intrinsic Teneurin Properties

In *Drosophila*, Teneurins mediate partner matching and synaptic organization at the same synapses (Hong et al., 2012; Mosca et al., 2012; Mosca and Luo, 2014), seemingly creating a paradox. How can synapses detect a difference between homo- and heterophilic Teneurin pairs and respond accordingly? Temporal strategies may be used: if homo- and heterophilic Teneurin interactions are not concurrently used (i.e., if partner matching finishes before synapse organization begins), elevated expression may be developmentally downregulated, leaving the basal levels. This, however, is unlikely, as elevated levels persist after partner matching and synapse formation (Hong et al., 2012; Mosca et al., 2012; Mosca and Luo, 2014). In vertebrate systems, though, Teneurin-2 expression is temporally regulated (Otaki and Firestein, 1999a, b), so this strategy remains a formal regulatory possibility. Another intriguing possibility is that the different binding partners that convey Teneurin signals to downstream cellular machinery are temporally regulated. Thus, the receptors themselves may be constantly at the membrane, but their effectors are only expressed at times consistent with either a partner matching or synaptic organization role. Identification of these downstream binding partners and careful study of their regulation will shed light on this possibility.

**Figure 3:**
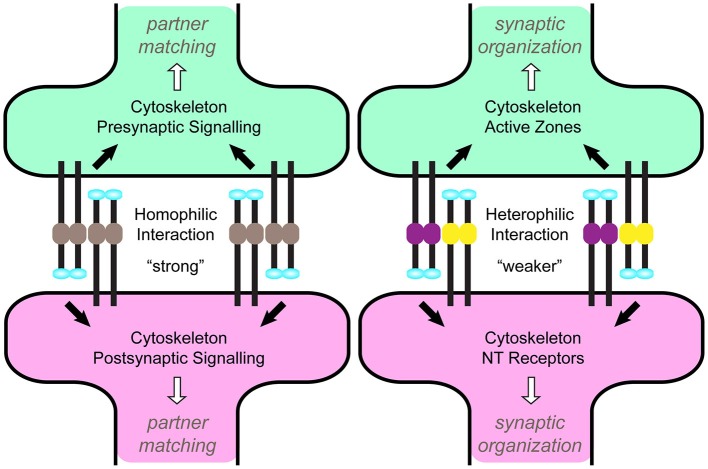
**A Model for homo- vs. heterophilic Teneurin signaling via tension**. A model (adapted from Beckmann et al., 2013) for how tension created by homophilic vs. heterophilic Teneurin interactions could distinguish partner matching from synaptic organization. The enhanced strength of homophilic interactions (left) alters cytoskeletal dynamics on the pre- and postsynaptic sides, activating signaling pathways that transition growth cones from exploring neurites to structures amenable to synapse formation. Weaker heterophilic interactions (right) regulate cytoskeletal organization and active zone apposition, leading to synaptic organization via signaling mechanisms distinct from partner matching.

An intriguing possibility may lie in the Teneurins themselves. If their intrinsic structural properties could distinguish homo- and heterophilic interactions, this would enable both signaling modes with the fewest restrictions (Figure 3). Biophysically, this may occur through tensile strength. Using atomic force microscopy and single-cell force spectroscopy, the tension between homo- and heterophilic Teneurin pairs has been recorded (Beckmann et al., 2013). Similar forces were required to break homophilic pairs and in all cases, exceeded those of heterophilic pairs. Homophilic strength also increased over time, depending on the intracellular domain. This presents an appealing model whereby different tensile strengths between homo- and heterophilic pairs could recruit different effectors or establish differing tensile networks, and mediate differential interactions for partner matching vs. synaptic organization. Such a difference may arise from domain asymmetry: in some (but not all) cases, the NHL domain mediates homophilic interaction (Beckmann et al., 2013), suggesting a more decentralized distinguishing mechanism. In all pairwise heterophilic comparisons (Figure 1), different domains exist to permit such a distinction. Some controversy exists, however, as an independent study suggested Teneurins could not support homophilic cell-cell adhesion (Boucard et al., 2014); additional confirmation will be needed to resolve this discrepancy. If true, such a role for tension is not without precedent. Membrane tension can mediate integrin signaling (Grashoff et al., 2010; Ferraris et al., 2014), tissue morphogenesis (Rauskolb et al., 2014), cell migration and adhesion (Parsons et al., 2010; Benson and Huntley, 2012; Cai et al., 2014), and vesicle dynamics (Siechen et al., 2009; Ahmed et al., 2012). These processes often function through altering cytoskeletal tension. As the Teneurins interact with the cytoskeleton (see above), this further positions them to regulate tension. Indeed, Teneurin-4 promotes neurite outgrowth with the focal adhesion kinase (FAK; Suzuki et al., 2014), which itself modulates tension via actin dynamics (Schober et al., 2007). The intracellular domains may further distinguish homo- and heterophilic signals. Differences in polyglycine or polyproline stretches and potential phosphorylation sites in the N-terminus (Minet et al., 1999; Tucker and Chiquet-Ehrismann, 2006) may contribute to differences in tension or in the identity of downstream interactors. Domain analysis *in vivo* will be able to offer important clarity on these differences are achieved.

## Teneurins in Human Disease: A Synaptopathic Origin?

In recent years, the synaptic basis of neurological disorders has been more concretely appreciated (Thompson and Luscher, 2014) as many intellectual disabilities have been associated with synaptic etiologies. Many psychiatric disorders are characterized by impaired synaptic function (Guilmatre et al., 2014). There is evidence for perturbed synaptic development (Clement et al., 2012), function (Földy et al., 2013; Rothwell et al., 2014), morphology (Südhof, 2008; Rothwell et al., 2014), elimination (Tsai et al., 2012b; Tang et al., 2014), and network homeostasis (Dickman and Davis, 2009; Fromer et al., 2014; Pocklington et al., 2014) in diverse intellectual disabilities (Zoghbi and Bear, 2012). These so-called “synaptopathies” including autism spectrum disorders (ASDs), bipolar disorder, and schizophrenia highlight the importance of ensuring correct synaptic organization to prevent such intellectual disabilities and neurodevelopmental disorders.

How may the Teneurins be synaptopathic? Teneurin mutations have been implicated in a number of intellectual disabilities. Large regions of the human X and 5th chromosomes containing Teneurins-1 and -2, respectively, are linked to mental retardation (Tucker and Chiquet-Ehrismann, 2006). Xq25 contains 12 genes and a number of potential microRNAs and noncoding elements that may be associated with X-linked mental retardation. Indeed, several variants of Teneurin-1 were identified in one ASD family (Nava et al., 2012), strengthening it as a candidate intellectual disability gene. Patients with mutations in X-linked regions also have severely impaired vision (Gustavson et al., 1993; Malmgren et al., 1993), consistent with known Teneurin roles in visual system patterning (Leamey and Sawatari, 2014). The case for Teneurin-2 is less clear: the 5q34 region associated with intellectual disabilities (Paoloni-Giacobino et al., 1999; Abuelo et al., 2000; Tucker and Chiquet-Ehrismann, 2006) contains at least 20 genes, including Teneurin-2 but also contains Slit3 and two GABA neurotransmitter receptors, genes with known roles in axon guidance and nervous system function. Though no clear association has been made for Teneurin-1 or Teneurin-2 and ASD, it is tempting to speculate a causal role. Teneurin-3 regulates eye development (Aldahmesh et al., 2012), optic nerve organization, and visual wiring (Leamey and Sawatari, 2014). These roles, though, are more reminiscent of roles in cellular morphogenesis (Kinel-Tahan et al., 2007), but a proper distinction regarding synaptic function still needs to be determined (Antinucci et al., 2013). Indeed, in all cases, failed wiring could also cause defective network regulation via impaired synaptic excitation, producing neural dysfunction similar to ASDs. Future work must be careful to determine whether potential involvement is related to their roles in partner matching or synaptic organization.

A tantalizing link between Teneurins and synaptopathies rests with bipolar disorder. Genome-wide association studies linked Teneurin-4 mutations to enhanced susceptibility to bipolar disorder (Psychiatric GWAS Consortium Bipolar Disorder Working Group, 2011; Georgi et al., 2014). Potential links also exist for Teneurin-2 (Cruceanu et al., 2013). Bipolar disorder is thought to be associated with defects in synaptic physiology and plasticity (Lopez de Lara et al., 2010; Du et al., 2011), resulting in improper circuit processing (Schloesser et al., 2008). How Teneurin-4 mutations enhance susceptibility to bipolar disorder is unknown, though in general, enhanced risks may be due to changes in reward processing in the amygdala (Heinrich et al., 2013). Circuit defects could also potentially arise from improper wiring due to impaired neurite outgrowth (Suzuki et al., 2014) or glial development (Suzuki et al., 2012), though Teneurin-4 associated myelination defects likely only have consequences for the peripheral nervous system. But as bipolar disorder can have a synaptic etiology, it is tempting to hypothesize that Teneurin-4-associated susceptibility can arise from synaptic defects. How might this occur? One favored mechanism is an improper regulation of intracellular signaling (Manji et al., 2003): factors like Protein Kinase C are targets of the most commonly used bipolar drugs and are intricately intertwined with cAMP and cGMP second messenger systems (Gould et al., 2004; Quiroz et al., 2004). While the Teneurins are not known to directly interact with such systems, their proper localization, as well as that of their regulating ion channels, relies on synaptic organization. A reasonable hypothesis states that the abrogation of such organization following Teneurin mutation would impair signaling, enhancing susceptibility to bipolar disorder. Alternatively (or concurrently), Teneurin-4 mutations may affect inhibitory GABAergic synapses. Considerable evidence suggests that GABAergic transmission is abnormal in bipolar patients (Benes, 2011). If Teneurin-4 mutations alter the array of inputs onto, or made by, GABAergic circuits, this could create a network more amenable to dysfunction. Such considerations should guide future work to understand whether patients who bear Teneurin-4 mutations demonstrate synaptic deficits (Wen et al., 2014) consistent with bipolar disorder. Expanding such analyses to the remaining Teneurins and comparing them to phenotypes associated with intellectual disabilities will greatly advance the search for a root cause.

## Conclusions

The Teneurins have emerged as transsynaptic, cell surface molecules essential for synaptic organization. In *Drosophila*, they order the underlying synaptic cytoskeleton and ensure proper synaptic function, differentiation, and morphology. Understanding their complete synaptic role, however, is in its infancy, and remains an exciting area for future study. Further work is needed to decipher the mechanisms of synaptic Teneurin function, leading to how they affect human brain function. Indeed, while human nervous systems have more complex circuit regulatory requirements than invertebrates, the marked conservation in worm, fly, and mouse models for Teneurin function suggests that their diverse roles may rely on similar core mechanisms. It will be critical to determine how these synaptic roles differ from those of partner matching. Understanding their downstream effectors and how homo- and heterophilic Teneurin signals are distinguished in partner matching vs. synaptic organization will achieve this goal. Linking these mechanistic studies to those of patients with Teneurin mutations will further enhance our understanding of how these proteins function. As future work addresses these questions, the Teneurins may take their place alongside known synaptopathic and ASD genes like Neurexin and Neuroligin as critical synaptic determinants, highlighting their importance in producing a functioning, organized brain.

## Conflict of Interest Statement

The author declares that the research was conducted in the absence of any commercial or financial relationships that could be construed as a potential conflict of interest.
